# Appearance of basal cell carcinoma on untreated capillary malformation^؆^

**DOI:** 10.1016/j.abd.2025.501190

**Published:** 2025-08-09

**Authors:** Catalina Buchroithner, Nelson Lobos, Gabriel Neely, Arturo Madrid, Valentina Darlic, Alex Castro

**Affiliations:** a Dermatology Department, Clínica Alemana, Universidad del Desarrollo, Santiago, Chile; b Dermato-Oncology Unit, Head and Neck Surgery Department, Instituto Nacional del Cancer, Santiago, Chile; c Head and Neck Surgery Department, Clínica Alemana, Universidad del Desarrollo, Santiago, Chile; d Pathology Department, Clínica Alemana, Universidad del Desarrollo, Santiago, Chile

Dear Editor,

Capillary malformations are congenital abnormalities in the morphogenesis of capillary vessels in the skin, consisting of a network of dilated blood vessels in the superficial dermis beneath a normal epidermis. They are present in 0.1%–0.3% of infants and represent the most common congenital vascular malformation.^1^ The development of basal cell carcinoma (BCC) within a capillary malformation is a rare finding, and its etiology remains unclear.^2^

We present the case of a 62-year-old female patient with a history of a left frontal nevus flammeus following the V1 trigeminal dermatome since birth, with no prior evaluation or treatment. She reported a seven-year history of an asymptomatic, progressively enlarging lesion in the left frontal region, accompanied by the development of nodules and tumors. On physical examination, an erythematous-violaceous plaque with superficial nodules, some hyperkeratotic and tending to coalesce, was observed (Fig. 1). The lesion had a soft consistency and followed the distribution of the V1 trigeminal branch. Dermoscopy revealed an erythematous base with ectatic red vessels, presenting as linear structures with a horizontal orientation and rounded, globular structures with a vertical orientation. Additionally, some nests and blue-gray globules were noted (Fig. 2). Doppler ultrasound identified a low-flow vascular malformation with poorly defined borders in the supraciliary and left frontal region, extending to the junction with the scalp. An incisional biopsy, guided by dermoscopic demarcation, revealed superficial hyperkeratosis, an infundibulum with cystic dilation, and irregular nests of basaloid cells infiltrating into the deep reticular dermis, consistent with pigmented nodular basal cell carcinoma (Fig. 3). A Limberg flap was selected to provide broader coverage without tissue tension and to facilitate closure of the secondary defect (Fig. 4).

**Figure 1:**
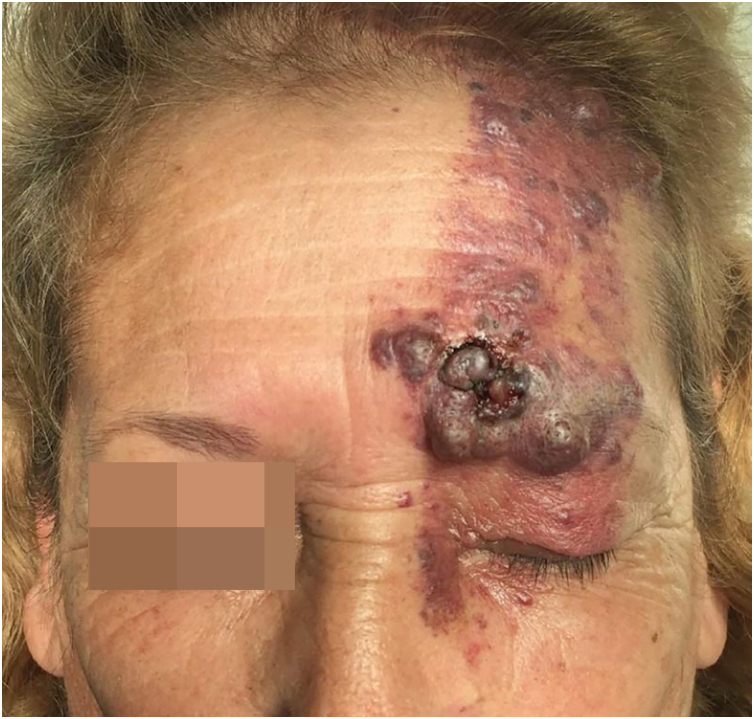
Erythematous-violaceous plaque with superficial nodules, some hyperkeratotic, with a tendency to coalesce, soft consistency, following the distribution of the V1 trigeminal branch.

**Figure 2:**
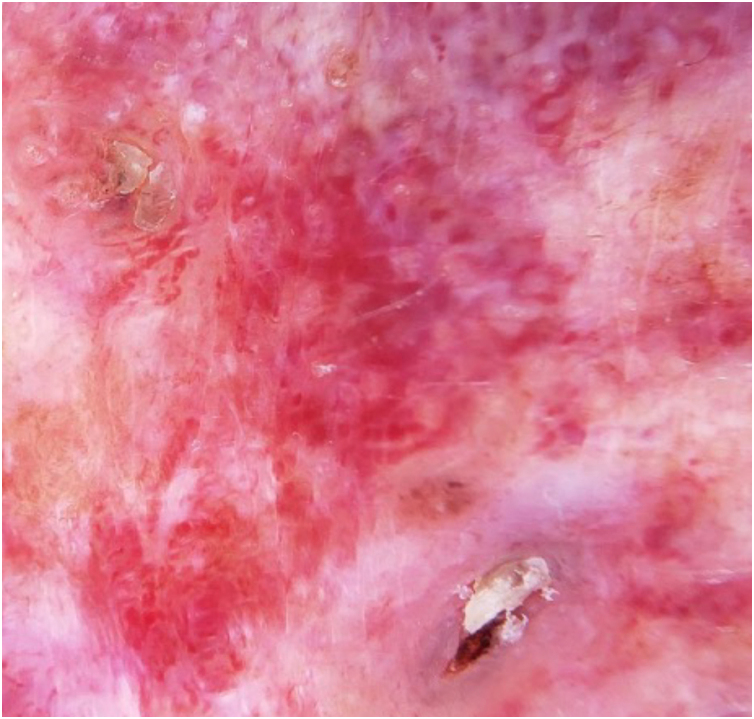
Dermoscopy image displays an erythematous base with ectatic red vessels, linear with a horizontal orientation and rounded, globular with a vertical orientation. Associated with some nests and blue-gray globules, rosettes, fibrosis, and areas resembling seborrheic keratosis.

**Figure 3:**
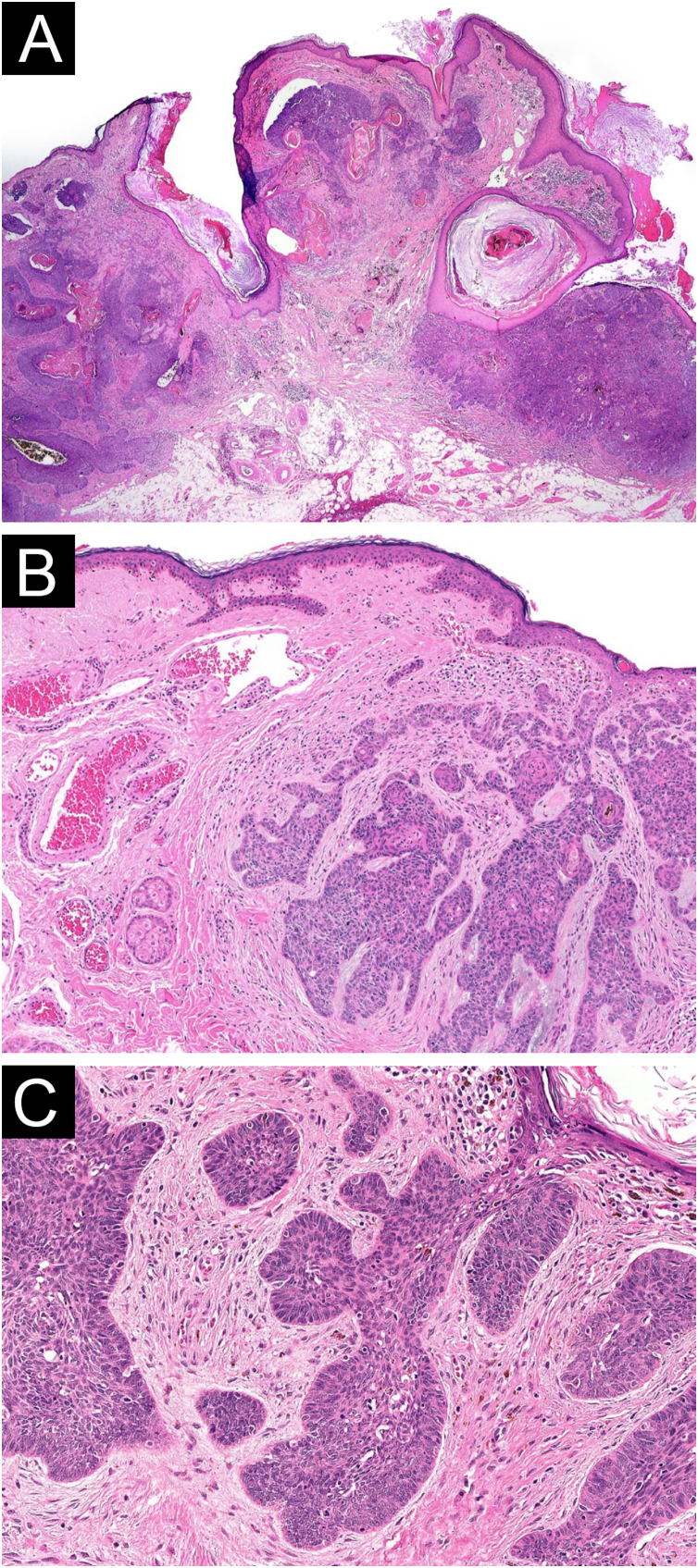
Histopathology (A) Hematoxylin & eosin, ×40: Low power shows tumor composed of nests of basaloid cells. Some retraction spaces are visible between tumor islands and stroma. (B) Hematoxylin & eosin, ×100: Tumor is made of basaloid cells arranged in cords and nests. The stroma is fibrous and exhibits mucinous change. At the left, abnormal dilated dermal vessels are seen. (C) Hematoxylin & eosin, ×200: High power shows tumor nests composed of basaloid cells with oval nuclei and scant cytoplasm, forming a peripheral palisading.

**Figure 4:**
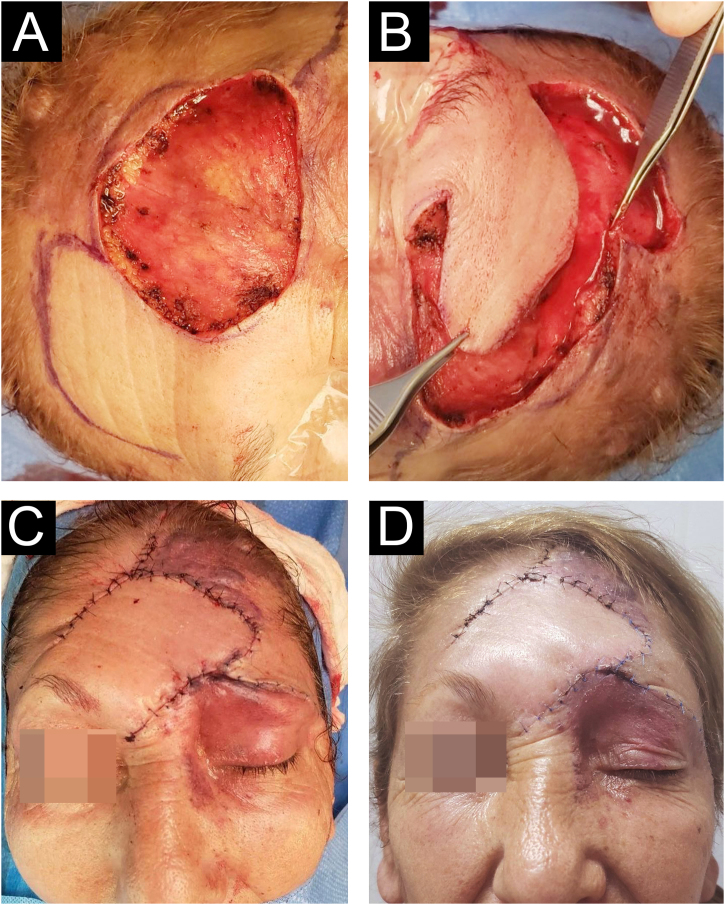
Limberg flap was chosen to achieve greater coverage without tissue tension and to facilitate the closure of the secondary defect. (A) Primary defect. (B) Closure planning. (C) Immediate postoperative period. (D) Postoperative day 21.

Previous therapy, particularly radiotherapy, has been proposed as a contributing factor to the development of BCCs within capillary malformations. Although this treatment approach is now obsolete for these lesions, its association with BCC has been reported in up to 75% of cases.^2^, ^3^ The first description of BCC arising in a capillary malformation was made by Scott in 1948,^4^ and to date, approximately 27 cases have been reported,^5^ of which only six occurred in untreated capillary malformations.^2^, ^6^ It has been suggested that the production of an oncogenic factor by the underlying static vessels may render the epidermis more susceptible to ultraviolet or ionizing radiation.^2^

In conclusion, we report a case of BCC arising within an untreated facial capillary malformation, an association that has been rarely documented and poses a diagnostic challenge. Dermoscopy plays a crucial role in highlighting vascular and pigmented features suggestive of basal cell carcinoma, particularly in this context. However, further studies are needed to determine the presence of local factors that may contribute to the development of these tumors within a capillary malformation.

## Financial support

None declared.

## Author's contributions

Catalina Buchroithner: Approval of the final version of the manuscript; critical literature review; manuscript critical review; preparation and writing of the manuscript.

Nelson Lobos: Approval of the final version of the manuscript; critical literature review; manuscript critical review; preparation and writing of the manuscript.

Gabriel Neely: Critical literature review; manuscript critical review; preparation and writing of the manuscript.

Arturo Madrid: Critical literature review; manuscript critical review; preparation and writing of the manuscript.

Valentina Darlic: Critical literature review; manuscript critical review; preparation and writing of the manuscript.

Alex Castro: Critical literature review; manuscript critical review; preparation and writing of the manuscript.

## Conflicts of interest

None declared.
